# A curated mammography data set for use in computer-aided detection and diagnosis research

**DOI:** 10.1038/sdata.2017.177

**Published:** 2017-12-19

**Authors:** Rebecca Sawyer Lee, Francisco Gimenez, Assaf Hoogi, Kanae Kawai Miyake, Mia Gorovoy, Daniel L. Rubin

**Affiliations:** 1 Biomedical Informatics Training Program, Stanford University, Stanford, CA 94305, USA; 2 Department of Radiology and Medicine (Biomedical Informatics Research), Stanford University, Stanford, CA 94305, USA; 3 Department of Radiology (Breast Imaging), Stanford University, Stanford, CA 94305, USA

**Keywords:** Radiography, Breast cancer

## Abstract

Published research results are difficult to replicate due to the lack of a standard evaluation data set in the area of decision support systems in mammography; most computer-aided diagnosis (CADx) and detection (CADe) algorithms for breast cancer in mammography are evaluated on private data sets or on unspecified subsets of public databases. This causes an inability to directly compare the performance of methods or to replicate prior results. We seek to resolve this substantial challenge by releasing an updated and standardized version of the Digital Database for Screening Mammography (DDSM) for evaluation of future CADx and CADe systems (sometimes referred to generally as CAD) research in mammography. Our data set, the CBIS-DDSM (Curated Breast Imaging Subset of DDSM), includes decompressed images, data selection and curation by trained mammographers, updated mass segmentation and bounding boxes, and pathologic diagnosis for training data, formatted similarly to modern computer vision data sets. The data set contains 753 calcification cases and 891 mass cases, providing a data-set size capable of analyzing decision support systems in mammography.

## Background & Summary

Computer-aided detection (CADe) and diagnosis (CADx) systems are designed to assist radiologists for mammography interpretation. CADe is employed to discover abnormal structures within the mammogram while CADx is used to determine the significance of the discovered abnormality. Despite promising results, current CADe systems are limited by high false-positive rates^1^, and CADx systems for mammography are not yet approved for clinical use^2^. Although the technical difficulty of CAD in mammography has been substantial, there is another obstacle that must be addressed to enable this research: decision support system evaluation.

Our review of the CAD literature reveals inconsistent data sources and data-set sizes. In addition, only few of the published results can be reproduced directly, as most evaluation data are not public. Tables 1 and 2 contain a sample of many systems, CADe and CADx, respectively, that have been evaluated using private data sets or undefined portions of public data sets. Without common data sets, it is impossible to rigorously compare methods. This is hindering CAD research in mammography. We seek to address this challenge by providing a standard data set for evaluation.

The non-medical computer vision community has adopted an open research approach. This includes provision of standard data sets for evaluation of algorithms. For example, ImageNet is a database of 14,197,122 images from 27 ‘high-level’ categories including animals, food, and vehicles. Each category has at least 51 sub-categories, allowing for highly specific classifications^3^. Other public databases include Mixed National Institute of Standards and Technology (MNIST) database, a database of hand-written digits^4^, and Caltech 256, a database of 265 object categories such as helicopters, planes, motorbikes, and school buses^5^. These data sets and others like them have provided a benchmark for computer vision research. Many researchers point to the existence of these open data sets as the primary drivers for recent successes in image classification technologies, such as deep learning.

Conversely, few well-curated public data sets have been provided for the mammography community. These include the Digital Database for Screening Mammography (DDSM)^6^, the Mammographic Imaging Analysis Society (MIAS) database^7^, and the Image Retrieval in Medical Applications (IRMA) project^8^. Although these public data sets are useful, they are limited in terms of data set size and accessibility. For example, most researchers using the DDSM do not leverage all its images for a variety of historical reasons. When the database was released in 1997, computational resources to process hundreds or thousands of images were not widely available. Additionally, the DDSM images are saved in non-standard compression files that require the use of decompression code that has not been updated or maintained for modern computers. Finally, the region-of-interest (ROI) annotations for the abnormalities in the DDSM were provided to indicate a general position of lesions, but not a precise segmentation for them. Therefore, many researchers must implement segmentation algorithms for accurate feature extraction.

While there are substantial challenges in using the DDSM for method evaluation, due to its size and other unique characteristics, we believe that it can still be a powerful resource for imaging research. The DDSM is a database of 2,620 scanned film mammography studies. It contains normal, benign, and malignant cases with verified pathology information. The scale of the database along with ground truth validation makes the DDSM a useful tool in the development and testing of decision support systems despite the fact that the images are scanned film instead of full field digital mammograms. This is because there is currently no mammography database of this size publicly available. We report here the development of, and propose to release, the CBIS-DDSM (Curated Breast Imaging Subset of DDSM), an updated version of the DDSM providing easily accessible data and improved ROI segmentation. This resource will contribute to the advancement of decision support system research in mammography, supplying a standardized mammography data.

## Methods

**Figure 1:**
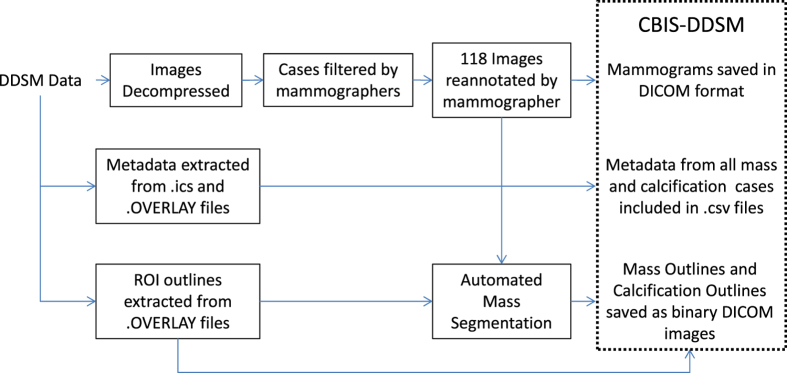
Flow diagram of preparation of CBIS-DDSM.

The DDSM already contains a large amount of information for each of its 2,620 cases. However, some information is limited, specifically the ROI annotations, while other information is difficult to access. We have solved these issues by updating the ROI segmentations and by gathering and reformatting the metadata into a more accessible format. Figure 1 shows a diagram of the processes performed to prepare the data set: image decompression and reannotation and metadata extraction and reformatting.

### Description of DDSM

The DDSM is a collection of mammograms from the following sources: Massachusetts General Hospital, Wake Forest University School of Medicine, Sacred Heart Hospital, and Washington University of St Louis School of Medicine. The DDSM was developed through a grant from the DOD Breast Cancer Research Program, US Army Research and Material Command, and the necessary patient consents were obtained by the original developers of the DDSM^6^. The cases are annotated with ROIs for calcifications and masses, as well as the following information that may be useful for CADe and CADx algorithms: Breast Imaging Reporting and Data System (BI-RADS) descriptors for mass shape, mass margin, calcification type, calcification distribution, and breast density; overall BI-RADS assessment from 0 to 5; rating of the subtlety of the abnormality from 1 to 5; and patient age.

### Parse semantic features

DDSM provides metadata in the form of.ics files. These files include the patient age, the date of the study, as well as the date of digitization, the dense tissue category, the scanner used to digitize, and the resolution of each image. Additionally, those cases with abnormalities have.OVERLAY files that contain information about each abnormality, including type of abnormality (mass or calcification) and the BI-RADS descriptors mentioned above. These metadata have been extracted and compiled into a single comma-separated values (CSV) file.

### Removal of questionable mass cases

It has been noted by other researchers that not all DDSM ROI annotations are accurate^9^, and we found that some annotations indicate suspicious lesions that are not seen in the image. Due to this issue, we acquired the assistance of a trained mammographer who reviewed the questionable cases. In this process, we found 339 images in which a mass was not clearly seen. These images were removed from the final data set. Additionally, several cases were removed by TCIA due to personal health information in the images.

### Image decompression

DDSM images are distributed as lossless joint photographic experts group (JPEG) files (LJPEG), an obsolete image format. The only library capable of decompressing these images is the Stanford PVRG-JPEG Codec v1.1, which was last updated in 1993. We modified the PVRG-JPEG codec to successfully compile on an OSX 10.10.5 (Yosemite) distribution using Apple GCC clang-602.0.53. The original decompression code outputs data in 8-bit or 16-bit raw binary bitmaps^6^. We wrote python tools to read this raw data and store it as 16-bit gray scale Tag Image File Format (TIFF) files. These files were later converted to Digital Imaging and Communications in Medicine (DICOM) format, which is standard for medical images. This process is entirely lossless and preserved all information from the original DDSM files.

### Image processing

The original DDSM files were distributed with a set of bash and C tools for Linux to perform image correction and metadata processing. These tools were very difficult to refactor for use on modern systems. Instead, we re-implemented these tools in Python to be cross-platform and easy to understand for modern users.

All images in the DDSM were derived from several different scanners at different institutions. The DDSM data descriptions provide methods to convert raw pixel data into 64-bit optical density values, which are standardized across all images. Optical density values were then re-mapped to 16-bit gray scale TIFF files and later converted to DICOM format for the data repository.

The DDSM automatically clips optical density values to be between 0.05 and 3.0 for noise reduction. We perform this clipping as well, but provide a flag to remove the clipping and retain the original optical density values.

### Image cropping

Several CAD tasks require only analyzing abnormalities (the portion of the image in the ROI) without needing the full mammogram image. We provide a set of convenience images, which are focused crops of abnormalities. Abnormalities were cropped by determining the bounding rectangle of the abnormality with respect to its ROI.

### Mass segmentation

**Figure 2:**
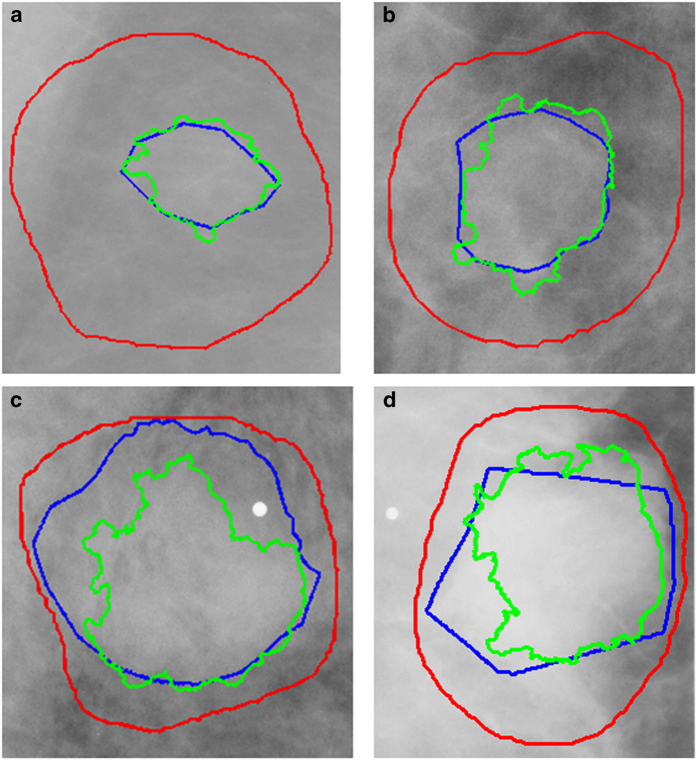
Example ROI outlines from each of the four BI-RADS density categories. The Dice’s coefficients are provided for each. (**a**) Density 1 ROI, *D*_*H,C*_=0.904, *D*_*H,D*_=0.237, (**b**) Density 2 ROI, *D*_*H,C*_=0.886, *D*_*H,D*_=0.423, (**c**) Density 3 ROI, *D*_*H,C*_=0.749, *D*_*H,D*_=0.797, (**d**) Density 4 ROI, *D*_*H,C*_=0.808, *D*_*H,D*_=0.682. Outlines: DDSM (red), hand-drawn (blue), automated (green).

Mass margin and shape have long been proven substantial indicators for diagnosis in mammography. Because of this, many methods are based on developing mathematical descriptions of the tumor outline. Due to the dependence of these methods on accurate ROI segmentation and the imprecise nature of many of the DDSM-provided annotations, as seen in Fig. 2, we applied a lesion segmentation algorithm (described below) that is initialized by the general original DDSM contours but is able to supply much more accurate ROIs. Figure 2 contains example ROIs from the DDSM, our mammographer, and the automated segmentation algorithm. As shown, the DDSM outlines provide only a general location and not a precise mass boundary. The segmentation algorithm was designed to provide exact delineation of the mass from the surrounding tissue. This segmentation was done only for masses and not calcifications.

Lesion segmentation was accomplished by applying a modification to the local level set framework as presented in Chan and Vese^10–12^. Level set models follow a non-parametric deformable model, thus can handle topological changes during evolution. Chan-Vese model is a region-based method that estimates spatial statistics of image regions and finds a minimal energy where the model best fits the image, resulting in convergence of the contour towards the desired object. Our modification of the local framework includes automated evaluation of the local region surrounding each contour point. For low contrast lesions, small local region is determined, and excessive curve evolution is thus prevented. On the other hand, for noisy or heterogeneous lesions, a relatively large local region is assigned to the contour point to prevent convergence of the level set contour into local minima. Local frameworks require an initialization of the contour, and thus in our case the original DDSM annotation was used as the level set segmentation initialization.

### Standardized train/test splits

**Figure 3:**
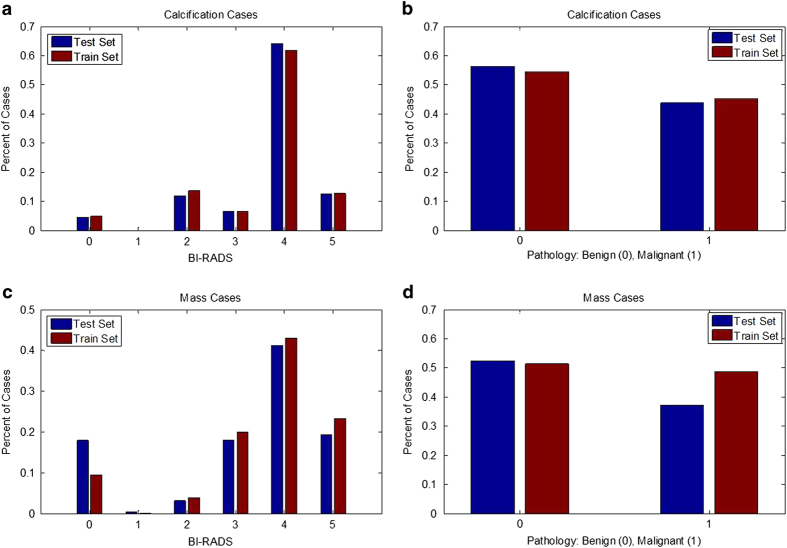
Histograms showing distribution of reading difficulty for training and test sets. Mass and calcification cases were split into training and test sets based on BI-RADS assessment. (**a**) Histogram of BIRADS for each abnormality in training and test sets with calcifications, (**b**) Histogram of benign and malignant cases for training and test sets with calcifications, (**c**) Histogram of BIRADS for each abnormality in training and test sets with masses, (**d**) Histogram of benign and malignant cases for training and test sets with masses.

Separate sets of cases for training and testing algorithms are important for ensuring that all researchers are using the same cases for these tasks. Specifically, the test set should contain cases of varying difficulty in order to ensure that the method is tested thoroughly. The data were split into a training set and a testing set based on the BI-RADS category. This allows for an appropriate stratification for researchers working on CADe as well as CADx. Note that many of the BI-RADS assessments likely were updated after additional information was gathered by the physician, as it is unconventional to subscribe BI-RADS 4 and 5 to screening images. The split was obtained using 20% of the cases for testing and the rest for training. The data were split for all mass cases and all calcification cases separately. Here ‘case’ is used to indicate a particular abnormality, seen on the craniocaudal (CC) and/or mediolateral oblique (MLO) views, which are the standard views for screening mammography. Figure 3 displays the histograms of BI-RADS assessment and pathology for the training and test sets for calcification cases and mass cases. As shown, the data split was performed in such a way to provide equal level of difficulty in the training and test sets. Table 3 contains the number of benign and malignant cases for each set.

### Code availability

The code used to generate these data sets from a raw dump of the DDSM was written in Python v2.7.9. It is publicly available as a git repository on GitHub at github.com/fjeg/ddsm_tools.

## Data Records

The images are distributed at the full mammography and abnormality level as DICOM files. Full mammography images include both MLO and CC views of the mammograms.

Abnormalities are represented as binary mask images of the same size as their associated mammograms. These mask images delineate the ROI of each abnormality. Users can perform an element-wise selection of pixels within an abnormality mask that was created for each mammogram. For convenience of abnormality analysis, we have also distributed images containing just the abnormalities cropped by the bounding box of their ROI. Abnormality files have been separated into Java Network Launch Protocol (JNLP) files based on abnormality type, training or test set, and image type (full mammogram, ROI mask, or cropped mammogram).

The following files contain the mammograms and ROIs for the cases with calcifications:

- Calc-Training_full_mammogram_images_1-doiJNLP-PrQ05L6k.jnlp
- Calc-Training_ROI-mask_and_crpped_images-doiJNLP-kTGQKqBk.jnlp
- Calc-Test_full_mammogram_images-doiJNLP-SiXj6kpS.jnlp
- Calc-Test_ROI-mask_and_crpped_images-doiJNLP-PsjCfTdf.jnlp

The following files contain the mammograms and ROIs for the cases with calcifications:

- Mass-Training_full_mammogram_images_1-doiJNLP-wv6aeYDn.jnlp
- Mass-Training_ROI-mask_and_crpped_images_1-doiJNLP-07gmVj4b.jnlp
- Mass-Test_full_mammogram_images-doiJNLP-6ccCrb8t.jnlp
- Mass-Test_ROI-mask_and_crpped_images-doiJNLP-SmEOyQFn.jnlp

Note that there is some overlap since some cases contain both calcifications and masses. Metadata for each abnormality is included as an associated CSV file containing the following:

- Patient ID: the first 7 characters of images in the case file
- Density category
- Breast: Left or Right
- View: CC or MLO
- Number of abnormality for the image (This is necessary as there are some cases containing multiple abnormalities.
- Mass shape (when applicable)
- Mass margin (when applicable)
- Calcification type (when applicable)
- Calcification distribution (when applicable)
- BI-RADS assessment
- Pathology: Benign, Benign without call-back, or Malignant
- Subtlety rating: Radiologists’ rating of difficulty in viewing the abnormality in the image
- Path to image files

There are individual files for mass and calcification training and test sets:

- mass_case_description_train_set.csv
- mass_case_description_test_set.csv
- calc_case_description_train_set.csv
- calc_case_description_test_set.csv

All these files are available via Data Citation 1.

## Technical Validation

The details of data collection may be found at the primary DDSM website6. We have improved the quality of this data set by distributing the data in a more accessible format, specifically as decompressed images and updated metadata extraction code, as well as by providing improved ROI segmentation and training and testing splits for evaluation. The methods for the validation of the ROI segmentation are given below.

### Segmentation evaluation

**Figure 4:**
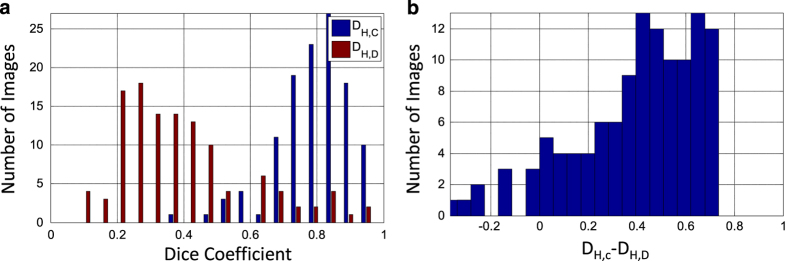
Results from comparison of new hand-drawn outlines with computer-generated and original DDSM outlines. (**a**) Histogram of Dice’s coefficients for new hand-drawn and computer-generated segmentation (*D*_*H,C*_) and new hand-drawn and DDSM (*D*_*H,D*_). The Dice’s coefficients were computed for 118 images. The mean *D*_*H,C*_ is 0.792±0.108, and the mean *D*_*H,D*_ is 0.398±0.195. (**b**) Histogram of *D*_*H,C*_−*D*_*H,D*_. The mean difference in Dice’s coefficient was 0.395±0.257. The Wilcoxon signed rank test between *D*_*H,C*_ and *D*_*H,D*_ yielded a *P*-value of 5.54×10−19.

Ideally, we would provide hand-drawn segmentations for each mass lesion in CBIS-DDSM. However, this would be a prohibitively large task to accomplish. We thus used an automated segmentation algorithm with the goal of providing better segmentations than those available in the DDSM. Since the segmentations we provide for the mass lesions in CBIS-DDSM were generated by our automated algorithm, we evaluated the segmentations in CBIS-DDSM by comparing ROIs from 118 images in CBIS-DDSM with hand-drawn outlines that were provided by an experienced radiologist. We computed the Dice coefficients between the computer and hand-drawn ROIs, *D*_*H,C*_. This was compared to the Dice coefficients between the original DDSM annotations and the newly hand-drawn annotations, *D*_*H,D*_. The Dice coefficient is a common metric for validity of image segmentation. Additionally, we use the Wilcoxon signed rank test to determine the statistical significance of *D*_*H,C*_ as compared to *D*_*H,D*_. We utilized this test instead of a standard *t*-test due to the non-normal distributions of Dice coefficients, as seen in Fig. 4. Finally, we examined the histogram and statistics of the difference between *D*_*H,C*_ and *D*_*H,D*_.

The average *D*_*H,C*_ was 0.792±0.108, and the average *D*_*H,D*_ was 0.398±0.195. Figure 4 shows the histogram of *D*_*H,C*_ and *D*_*H,D*_ for each of the 118 new hand-drawn images. The histogram shows that the majority of the images were automatically segmented with high correlation to the hand-drawn ROIs. The Wilcoxon signed rank test between *D*_*H,C*_ and *D*_*H,D*_ yielded a *P*-value of 5.54×0^−19^. The histogram in Fig. 4 shows the difference between *D*_*H,C*_ and *D*_*H,D*_. The mean difference in Dice’s coefficient was 0.395±0.257.

Figure 2 contains example ROIs from each of the BI-RADS density categories represented in the data set: (1) almost entirely fat, (2) containing scattered areas of fibroglandular density, (3) heterogeneously dense, and (4) extremely dense. The red outline indicates the original DDSM ROI, the blue is the new hand-drawn ROI, and the green is the automatically segmented ROI. The *D*_*H,C*_ for each of these ROIs with respect to the newly hand-drawn annotations were 0.904, 0.886, 0.749, and 0.808, and the *D*_*H,D*_ were 0.237, 0.423, 0.797, and 0.682, respectively. As expected, the accuracy of the computer method decreases with increase in breast density. Though *D*_*H,C*_ and *D*_*H,D*_ are comparable in some cases, particularly in higher density cases, the computer-generated ROIs were in much higher agreement with the hand-drawn ROIs and are overall much better segmentations.

## Additional information

**How to cite this article:** Lee, R. S. *et al.* A curated mammography data set for use in computer-aided detection and diagnosis research. *Sci. Data* 4:170177 doi: 10.1038/sdata.2017.177 (2017).

**Publisher’s note:** Springer Nature remains neutral with regard to jurisdictional claims in published maps and institutional affiliations.

## Supplementary Material



## Figures and Tables

**Table 1:** Sample Set of CADe Systems Reported in the Literature.

**Performance statistics of selected CADe methods for the detection of abnormalities**					
**Authors**	**Size of Data set (Cases)**	**Public or Private Data**	**Accuracy**	**Sensitivity**	**False Positives Per Image**
Karssemeijer and te Brake^13^	50	Public (MIAS*)	NA	90%	1
Mudigonda *et al.*^14^	56	Public (MIAS*)	NA	81%	2.2
Liu *et al.*^15^	38	Public (MIAS*)	NA	90%	1
Li *et al.*^16^	94	Private	NA	91%	3.21
Baum *et al.*^17^	63	Private	NA	89%	0.61
Kim *et al.*^18^	83	Private	NA	96%	0.2
Yang *et al.*^19^	203	Private	96.1%	95–98%	1.8
The *et al.*^20^	123	Private	NA	94%	2.3 per case
Sadaf *et al.*^21^	127	Private	NA	91%	NA
Chu *et al.*^22^	230	Public (DDSM†)	NA	98.5%	0.84

*Mammographic Imaging Analysis Society.

^†^Digital Database for Screening Mammography.

**Table 2:** Sample Set of CADx Systems Reported in the Literature.

**Performance statistics of selected CADx methods for the classification of masses**				
**Authors**	**Size of Data set (Cases)**	**Public or Private Data**	**Classification Accuracy**	Az*
Brzakovic *et al.*^23^	25	Private	85%	NA
Huo *et al.*^24^	65	Private	NA	0.94
Rangayyan *et al.*^25^	54	Public (MIAS†) and Private	91%	NA
Mudigonda *et al.*^26^	39	Public (MIAS†)	82.1%	0.85
Sahiner *et al.*^27^	102	Private	NA	0.91
Timp *et al.*^28^	465	Private	NA	0.77
Ganesan *et al.*^29^	282	Private	88.8%	NA
Görgel *et al.*^30^	78, 65	Private, Public (MIAS†)	91.4%, 90.1%	NA
Qiu *et al.*^31^	560	Private	77.14%	0.81
Choi *et al.*^32^	600	Public (DDSM‡)	NA	0.88

*Area under the receiver-operator characteristic curve.

^†^Mammographic Imaging Analysis Society.

^‡^Digital Database for Screening Mammography.

**Table 3:** Number of Cases and Abnormalities in the Training and Test Sets.

	**Benign Cases**	**Malignant Cases**
Calcification Training Set	329 cases (552 abnormalities)	273 cases (304 abnormalities)
Calcification Test Set	85 cases (112 abnormalities)	66 cases (77 abnormalities)
Mass Training Set	355 cases (387 abnormalities)	336 cases (361 abnormalities)
Mass Test Set	117 cases (135 abnormalities)	83 cases (87 abnormalities)
These numbers are different as some cases contain more than one abnormality.		
